# Supplementary material for: Reduced thermal expansion by surface-mounted nanoparticles in a pillared-layered metal-organic framework
Source: Commun Chem. 2022 Dec 22;5:177. doi: 10.1038/s42004-022-00793-2 (PMC9814677; doi:10.1038/s42004-022-00793-2)
Supplement: Supplementary file 2 — Supplementary Information [file 42004_2022_793_MOESM2_ESM.pdf]

Supplementary Information

Reduced Thermal Expansion by Surface-Mounted Nanoparticles in a Pillared-Layered Metal-Organic Framework

Jan Berger,[1] Alper-Sedat Dönmez,[1] Aladin Ullrich,[2] Hana Bunzen,[2] Roland A. Fischer[1] and Gregor Kieslich[1]**

[1]Inorganic and Metal-Organic Chemistry, Technical University of Munich, Lichtenbergstr. 4, 85748 Garching, Germany

[2]Institute of Physics, University of Augsburg, Universitätsstr. 1, 86159 Augsburg, Germany

Supplementary Notes

S1 Supplementary Methods 3

S1.1 General remarks 3

S1.2 Linker synthesis 3

S1.3 MOF and Pt@MOF synthesis 4

S1.4 MOF structure and flexibility 5

S2 Powder X-ray diffraction 6

S3 Sorption isotherms 7

S4 Thermogravimetric analysis and differential scanning calorimetry 8

S5 Variable temperature powder X-ray diffraction 9

S6 Pawley fitting and cell parameter 11

S7 Thermal expansion calculation 15

S8 Electron microscopy 16

S9 Supplementary references 20

S1 Supplementary Methods

S1.1 General remarks

All chemicals were purchased from commercial suppliers and used without further purification. MOF synthesis was conducted with pure N,N-dimethylformamide (DMF) (99.8%). Fresh DMF was used for solvent exchange. Ethanol for solvent exchange was purchased as technical grade and redistilled prior use. All experiments and procedures were carried out on air unless stated otherwise.

Liquid state NMR spectra were recorded on a Bruker Ultrashield DRX400 spectrometer (^{1}H: 400.13 MHz) at ambient temperature (298 K). The ^{1}H NMR spectroscopic chemical shifts δ are reported in ppm relative to tetramethylsilane. ^{1}H NMR spectra are referenced against the residual proton resonances of the respective deuterated solvent as an internal standard (DMSO-d_6: δ (1H) = 2.50 ppm). MOF samples were digested and subsequently measured in 0.5 ml DMSO-d_6 with 0.05 ml DCl (7.6 N), all other substances were dissolved and measured in DMSO-d_6.

S1.2 Linker synthesis

Organic linkers were synthesized via Williamson Etherification according to literature known procedures,[1] albeit slightly altered/optimized.

Dimethyl-2,5-dihydroxy-1,4-benzenedicarboxylate

2,5-Dihydroxyterephthalic acid (6.0 g, 30.3 mmol) is dispersed in MeOH (180 mL) and H_2SO_4 (98%, 5 mL, 94 mmol) is added dropwise at room temperature while stirring and the reaction mixture is refluxed for 12 h at 75 °C. The precipitate is subsequently filtrated and thoroughly washed with distilled water. After removing residual solvent *in vacuo* at 70 °C, the product is obtained as yellow powder (6.63 g, 28.9 mmol, 95 %).

^{1}H-NMR (400.13 MHz, DMSO-d_6) δ (ppm) = 9.82 (s, 2H, COOH), 7.26 (s, 2H, H_{Ar}), 3.86 (s, 6H, CH_3).

2,5-Dipropoxy-1,4-benzenedicarboxylic acid (H_2DPbdc)

Dimethyl-2,5-dihydroxy-1,4-benzenedicarboxylate (2.00 g, 8.84 mmol, 1.0 eq.) and dry K_2CO_3 (3.00 g, 21.7 mmol, 2.5 eq.) are suspended in DMF (150 mL). 1-Bromopropane (2.41 mL, 26.5 mmol, 3 eq.) is added dropwise while stirring. The reaction mixture is refluxed overnight at 75 °C, the solvent is subsequently removed at 70 °C under reduced pressure (5 mbar). A solution of KOH (1.74 g, 30.5 mmol, 3.5 eq.) in H_2O (250 mL) is added to the residue. The reaction mixture is again refluxed overnight at 110 °C. After cooling to room temperature HCl (10%) is added dropwise until the carboxylic acid is completely precipitated. The solid is filtered and thoroughly washed with distilled water. After removing residual water *in vacuo* at 70 °C, the product (2.15 g, 4.27 mmol, 86 %) is obtained as white/slightly beige powder.

^{1}H-NMR (400.13 MHz, DMSO-d_6) δ (ppm) = 12.92 (s, 2H, COOH), 7.26 (s, 2H, H_{Ar}), 3.95 (t, J = 6.4 Hz, 4H, O-CH_2), 1.78 - 1.62 (m, 4H, CH_2), 0.97 (t, J = 7.4 Hz, 6H, CH_3).

S1.3 MOF and Pt@MOF synthesis

General procedure for Zn_2(DP-bdc)$_2$dabco and Pt@Zn_2(DP-bdc)$_2$dabco

$Zn(NO_3)_2 \cdot 6\ H2O$ (89 mg, 0.30 mmol, 1 eq.), 1,4-diazabicyclo[2.2.2]octane (dabco) (34 mg, 0.30 mmol, 1 eq), the 2,5-dipropoxy-1,4-benzenedicarboxylic acid (H_2DPbdc; 85 mg, 0.30 mmol, 1 eq.) and the corresponding amount of potassium tetrachloridoplatinate (see Supplementary Table 1) are suspended in dimethyl formamide (DMF) (10 ml) in a 20 ml screw cap vial. The mixture is sonicated until complete suspension and absence of sedimentation (3-10 min), then heated in an oven for 24 or 48 h (see Supplementary Table 1). After cooling to room temperature, the formed crystalline white to light grey powder is washed and activated.

Washing and activation procedure

Washing and solvent exchange steps are conducted by vigorously shaking solid and solvent in a capped centrifuge tube, then centrifugation and decanting of the supernatant.

The obtained powder is washed immediately with DMF (3x 10 ml) until the supernatant is clear to yield the MOF material in its as-synthesized (**as**) state. While wet, this state is verified by X-ray powder diffraction (see chapter S2).

To remove DMF the **as** material is first washed with ethanol (10 ml), then twice soaked in fresh ethanol (2x 10 ml) overnight, and lastly soaked once in dichloromethane (10 ml) overnight. The wet powder is then pre-dried on air at ambient conditions before proper activation *in vacuo* at r.t. for 16 h and subsequently at 70 °C for 4 h. The resulting **dry** sample is stored under argon for further use to rule out possibility of gradual hydrolysis during prolonged exposure to ambient moisture.

Supplementary Table 1: Platinum precursor amounts and reaction conditions during MOF crystallization procedures.

ID	K_2PtCl_4 [mg]	temperature [°C]	heating time [h]
**D0**	none	120	48
**D1**	4.2	100	24
**D2**	10.4	120	24
**D3**	10.4	100	24

Linker and pillar incorporation ratio was verified via ^{1}H NMR (see below for specifications), CHNS contents were determined via combustion analysis. Zn and Pt contents were determined by atom adsorption spectroscopy or photometry for samples with more than 50 mg available with uncertainty of +- 0.1 w%. Elemental analysis was provided and conducted by the TUM CRC Microanalytical laboratory.

[Zn_2(DP-bdc)$_2$dabco – D0 (reference material)] ^{1}H NMR (400.13 MHz, DMSO-*d6*) δ (ppm) = 7.18 (s, 4H, H_{Ar}), 3.87 (t, J = 6.6 Hz, 8H, O-CH_2), 3.54 (s, 12H, dabco), 1.61 (h, J = 7.2 Hz, 8H, CH_2), 0.89 (t, J = 7.3 Hz, 12H, CH_3). **Elemental analysis:** calculated C 50.83, H 5.52, N 3.49, O 23.89, Zn 16.27; found C 50.59, H 5.41, N 3.67, Zn 16.19;

[Pt@Zn_2(DP-bdc)$_2$dabco – D1] ^{1}H NMR (400.13 MHz, DMSO-*d6*) δ (ppm) = 7.10 (s, 4H, H_{Ar}), 3.79 (t, J = 6.3 Hz, 8H, O-CH_2), 3.54 (s, 12H, dabco), 1.52 (h, J = 7.0 Hz, 8H, CH_2), 0.78 (t, J = 7.3 Hz, 12H, CH_3). **Elemental analysis:** found C 49.67, H 5.38, N 3.50, Zn 15.7, Pt 1.4;

[Pt@Zn$_2$(DP-bdc)$_2$dabco – D2] ^{1}**H NMR** (400.13 MHz, DMSO-*d*6) δ (ppm) = 7.09 (s, 4H, H_{Ar}), 3.78 (t, *J* = 6.3 Hz, 8H, O-CH_2), 3.53 (s, 12H, dabco), 1.52 (q, *J* = 6.9 Hz, 8H, CH_2), 0.78 (t, *J* = 7.4 Hz, 12H, CH_3). **Elemental analysis:** found C 49.67, H 5.38, N 3.50, Zn 15.7, Pt 1.9;

[Pt@Zn$_2$(DP-bdc)$_2$dabco – D3] ^{1}**H NMR** (400.13 MHz, DMSO-*d*6) δ (ppm) = 7.10 (s, 4H, H_{Ar}), 3.79 (t, *J* = 6.2 Hz, 8H, O-CH_2), 3.54 (s, 12H, dabco), 1.51 (p, *J* = 7.2 Hz, 8H, CH_2), 0.78 (t, *J* = 7.3 Hz, 12H, CH_3). **Elemental analysis:** found C 49.62, H 5.38, N 3.65, Zn 15.1, Pt 3.1;

S1.4 MOF structure and flexibility

Zn_2(DP-bdc)$_2$dabco is a pillared-layered MOF consisting of two-dimensional square-lattice layers spanned by paddlewheel units and DP-bdc^{2-} linkers which are congruently stacked and connected via the dabco pillars in the third dimension. Many derivatives of the Zn_2(fu-bdc)$_2$dabco family (fu = 2,5-functionalization) are flexible MOFs which can transition between a contracted **np** phase and an expanded **lp** phase (in a first-order phase transition).[2] The in this work studied MOF only undergoes this transition in response to polar solvents (like DMF, EtOH) and CO_2. Other functionalizations, however, unlock this flexibility in response to temperature or mechanical pressure as well.[3,4] These MOFs are synthesized in their **lp** state (due to the reaction medium DMF) and transitioned to their **np** phase during careful solvent exchange and removal. This state is metastable until exposure to abovementioned polar adsorbates which switch the MOF to its **lp** phase. This is then again reversible by i.e. solvent evaporation, drying, gas removal etc. The interested reader is directed to this review article on flexible MOFs.[5]

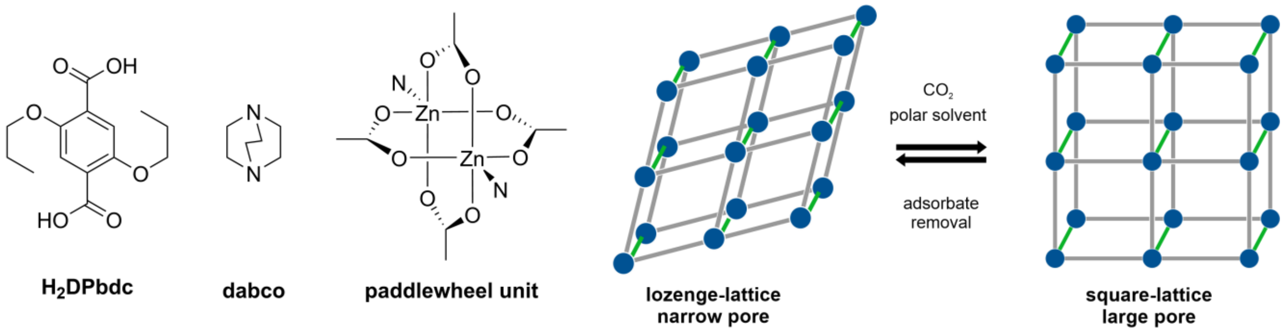

Supplementary Figure 1: From left to right structure of the terephthalic acid derivative linker, dabco pillar, and Zn paddlewheel building block. Only coordinating nitrogen atoms shown in the paddlewheel unit for clarity. Right hand structures visualize the np to lp phase transition as is present and triggered in Zn_2(DP-bdc)$_2$dabco by polar solvents (like DMF, EtOH) and CO_2. Blue nodes = paddlewheel unit, grey struts = DP-bdc^{2-}, green struts = dabco.

S2 Powder X-ray diffraction

PXRDs of the as-synthesized (**as**) and resolvated samples were measured using Bragg-Bentano geometry with a silicon wafer plate on a Rigaku Benchtop MiniFlex 600-C. X-ray Cu Kα radiation (λ = 1.5406 Å) with a voltage of 40 kV and current of 15 mA was used.

Activated (**dry**) samples and samples after the CO_2 and N_2 physisorption cycles were filled in glass capillaries in a glovebox under argon atmosphere and PXRDs were measured in Debeye-Scherrer geometry on a PANalytical Empyrean diffractometer. X-ray Cu Kα radiation (λ = 1.5406 Å) with a voltage of 45 kV and current of 40 mA was used.

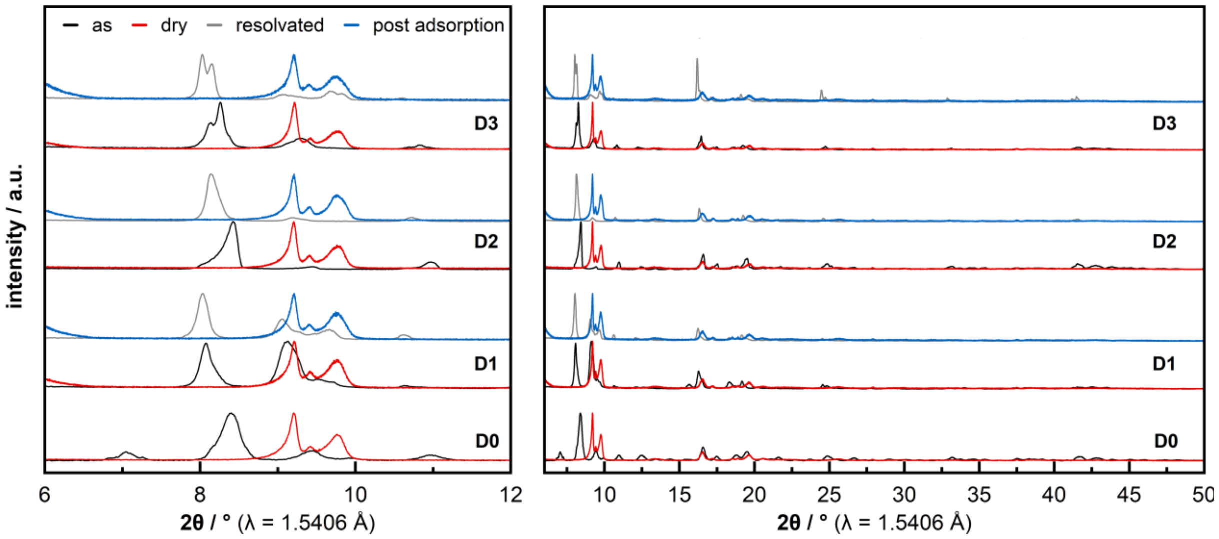

Supplementary Figure 2: PXRD patterns in the range of 2θ = 6-12° and 6-50°. Black: as-synthesized (**as**) with infiltrated DMF; red: activated (**dry**); grey: re-infiltrated with DMF after activation; blue: material state after all conducted gas adsorption measurements presented in this work (N_2 and CO_2). Transition from **lp** state in **as** to **np** state in **dry** is observable by shift of the 110 reflection from 2θ = 8.2-8.4° to 9.7° and intensity increase of the 001 reflection at 2θ = 9.2°.[6] Due to the very low NP loading and significant peak broadening coming from diffraction domains in the nano regime, no peaks corresponding to Pt nanoparticles can be observed at the expected angles around 40° (I(111)) and 47° (I(200)) 2θ.[7]

S3 Sorption isotherms

Adsorption measurements with N_2 (>99.999 vol%) at 77 K and CO_2 (>99.995 vol%) at 195 K were carried out on a 3Flex Physisorption from Micromeritics Instrument Corp., which uses a manometric method to determine the amount adsorbed at an equilibrated gas pressure.

Activated samples were transferred under dry argon atmosphere into preweighed sample tubes and capped with Micromeritics CheckSeals. Samples were subsequently activated again at 80 °C for 5 h under dynamic vacuum of ca. $1x10^{-3}$ mbar using a SmartVacPrep by Micromeritics Instrument Corp. to ensure absence of unwanted adsorbates and identical pre-measurement states of all samples. The mass of the adsorbent was then recorded, generally in the range of 50-70 mg. To facilitate proper degassing in context of the highly diffusion hindering pore environment, prior to each measurement samples were evacuated in situ at 80 °C for at least 20 h under dynamic vacuum of ca. $1x10^{-5}$ mbar. Free space of the sample tube was determined after measuring each adsorption isotherm using helium (>99.999 vol%). A liquid nitrogen bath was used for measurements at 77 K and a dry ice - acetone bath was used for measurements at 195 K.

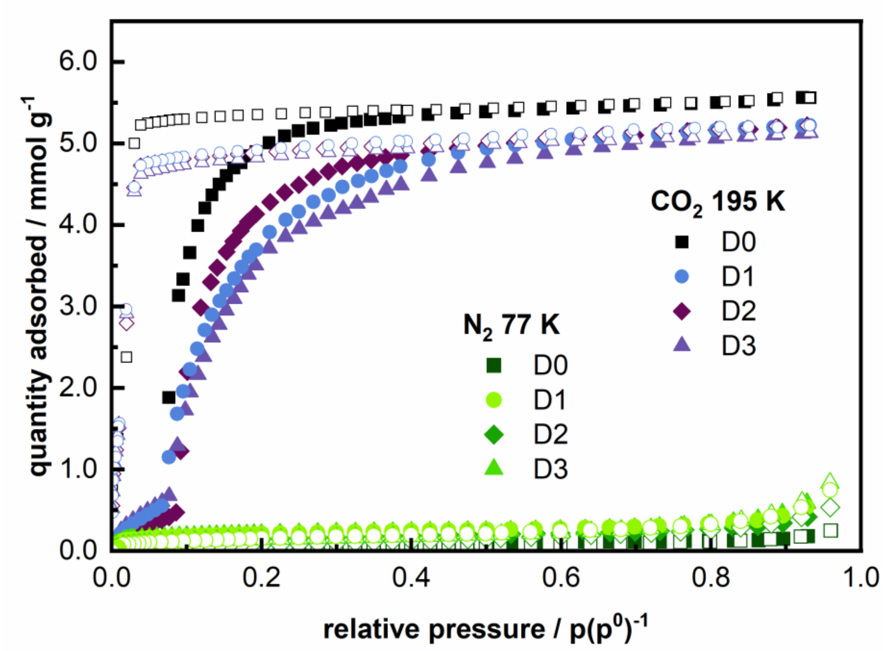

Supplementary Figure 3: Isotherms of N_2 at 77 K (green) and CO_2 at 195 K (blue/purple) on all materials with adsorption (filled symbols) and desorption (empty symbols), respectively. All materials with platinum nanoparticles (**D1-3**) show slightly decreased total absolute uptake compared to the reference material (**D0**), which we attribute to a small loss in accessible porosity due to nanoparticles blocking pores or pore access, and a widened **np** to **lp** transition pressure range. This more gradual opening is attributed to surface stress exhibited by the NPs which slightly rigidify the underlying framework. Layers around the NPs do as a result require a higher CO_2 partial pressure to undergo the opening breathing motion. This could explain both the widening of the opening pressure range, as well as the almost identical onset of the step. The widening is correlated to Pt nanoparticle presence, but not linearly to Pt content. The reverse **lp** to **np** transition during desorption occurs in line with **D0**. After desorption framework integrity and completed **np** phase transition of all materials was confirmed by PXRD (see section S2).

S4 Thermogravimetric analysis and differential scanning calorimetry

Thermogravimetric analysis coupled with differential scanning calorimetry (TGA-DSC) was conducted on a Netzsch TG-DSC STA 449 F5 in a temperature range from 25 °C to 800 °C with a heating rate of 10 K min^{-1} under argon flow (flow rate: 20 mL min^{-1}). It should be noted that the sample is briefly (few seconds) exposed to air before the measurement when the aluminium oxide pan is transferred from the argon filled transport vial to the sample holder stage.

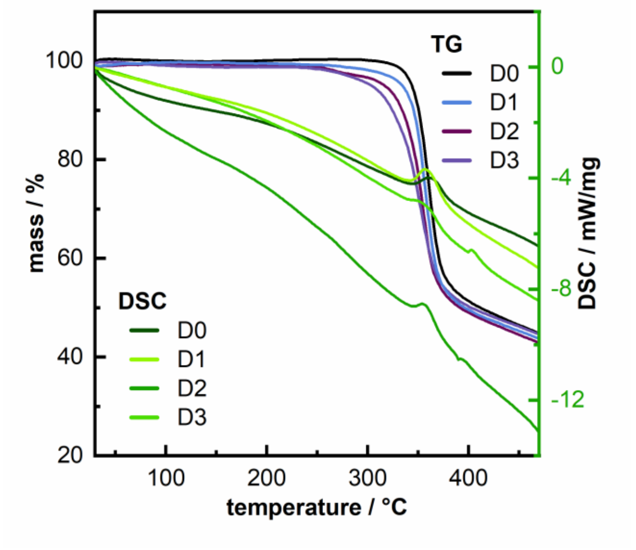

Supplementary Figure 4: TGA-DSC of all materials.

S5 Variable temperature powder X-ray diffraction

Activated **dry** samples were filled in borosilicate glass capillaries in a glovebox under argon atmosphere. Sealed capillaries were inserted into an open 1 mm diameter quartz capillary and measured in Debeye-Scherrer geometry from 323 K to 513 K to 323 K using a STOE INSITU HT2 Furnace on a STOE STADI P Dual. X-ray Mo Kα radiation (λ = 0.7107 Å) with a voltage of 50 kV and current of 40 mA was used.

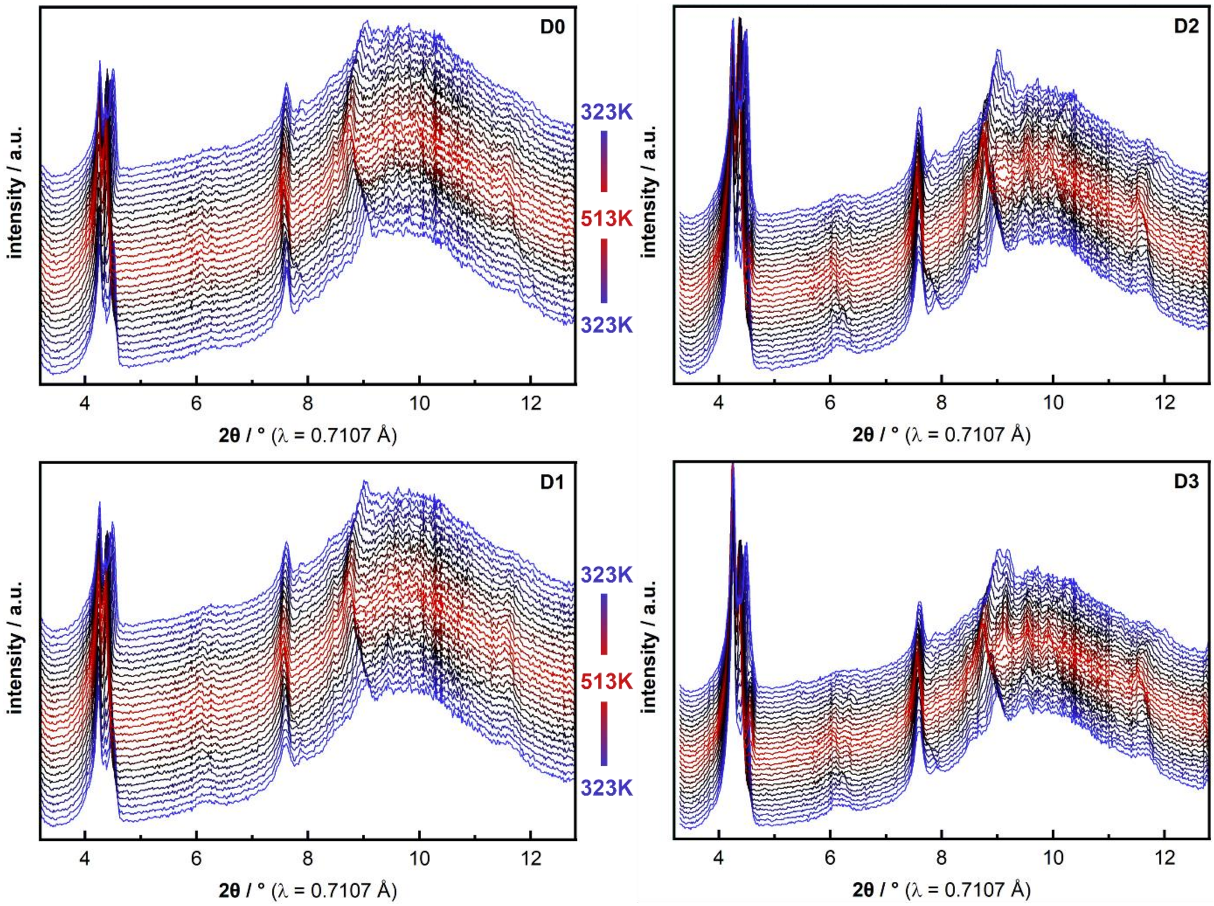

Supplementary Figure 5. Uncorrected VTPXRD patterns of dry **D0** (top left), **D1** (bottom left), **D2** (top right), and **D3** (bottom right) measured from 323K to 513K to 323K (temperature steps = 20K below 443K; 10K above 443K). The notable background is attributed to the secondary quartz glass capillary and polymeric windows of the oven sample stage (as verified by blank measurements). All materials show pronounced anisotropic thermal expansion of the **np** phase.

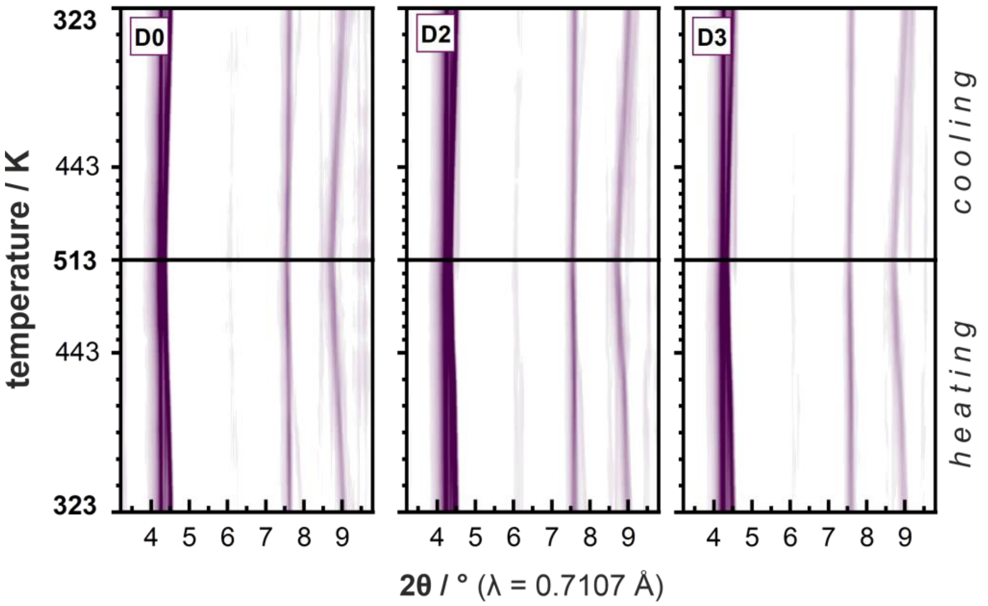

Supplementary Figure 6: Contour plot of VTPXRD data of **D0, D2,** and **D3** in the range of 2θ = 3.2 - 9.8. Darker purple corresponds to higher reflection intensity. Each set of 28 patterns is normalized to its highest intensity signal. Prominent gradual shift of peaks composites to lower angles is attributed to the literature known thermal expansion of $Zn_2(DP\text{-}bdc)_2$dabco.

S6 Pawley fitting and cell parameter

Raw VTXRD pattern (see chapter **S5**) were fitted directly.

Supplementary Table 2. Results of the Pawley fitting of the VTPXRDs recorded for **D0** (*h* = heating branch, *c* = cooling branch). Errors for given values a, b, c, β, and V correspond to last significant position. Space group was fixed for fitting according to literature known parent material property.

T [K]	space group	r_{wp}	a [Å]	b [Å]	c [Å]	α [°]	β [°]	γ [°]	V [Å^3]	branch
**323**	$P2_1/m$	1.67	18.468	10.166	9.444	90	93.53	90	1770	*h*
**343**	$P2_1/m$	1.51	18.437	10.202	9.451	90	93.67	90	1774	*h*
**363**	$P2_1/m$	1.66	18.435	10.250	9.453	90	93.74	90	1783	*h*
**383**	$P2_1/m$	1.65	18.423	10.302	9.464	90	93.87	90	1792	*h*
**403**	$P2_1/m$	1.62	18.390	10.355	9.468	90	94.11	90	1798	*h*
**423**	$P2_1/m$	1.50	18.361	10.408	9.473	90	94.37	90	1805	*h*
**443**	$P2_1/m$	1.50	18.388	10.451	9.481	90	94.29	90	1817	*h*
**453**	$P2_1/m$	1.52	18.416	10.478	9.483	90	94.14	90	1825	*h*
**463**	$P2_1/m$	1.51	18.464	10.502	9.490	90	94.26	90	1835	*h*
**473**	$P2_1/m$	1.51	18.480	10.516	9.495	90	94.20	90	1840	*h*
**483**	$P2_1/m$	1.67	18.513	10.521	9.498	90	94.23	90	1845	*h*
**493**	$P2_1/m$	1.50	18.530	10.527	9.498	90	94.27	90	1848	*h*
**503**	$P2_1/m$	1.47	18.544	10.540	9.500	90	94.33	90	1852	*h*
**513**	$P2_1/m$	1.47	18.559	10.538	9.502	90	94.34	90	1853	*h*
**503**	$P2_1/m$	1.50	18.550	10.546	9.499	90	94.34	90	1853	*c*
**493**	$P2_1/m$	1.37	18.525	10.537	9.496	90	94.35	90	1848	*c*
**483**	$P2_1/m$	1.46	18.514	10.529	9.495	90	94.35	90	1846	*c*
**473**	$P2_1/m$	1.44	18.492	10.520	9.495	90	94.41	90	1842	*c*
**463**	$P2_1/m$	1.46	18.472	10.505	9.491	90	94.39	90	1836	*c*
**453**	$P2_1/m$	1.46	18.447	10.485	9.490	90	94.38	90	1830	*c*
**443**	$P2_1/m$	1.57	18.430	10.467	9.486	90	94.44	90	1824	*c*
**423**	$P2_1/m$	1.51	18.379	10.421	9.479	90	94.52	90	1810	*c*
**403**	$P2_1/m$	1.52	18.377	10.379	9.474	90	94.41	90	1802	*c*
**383**	$P2_1/m$	1.64	18.394	10.330	9.460	90	94.29	90	1792	*c*
**363**	$P2_1/m$	1.70	18.397	10.279	9.453	90	94.13	90	1783	*c*
**343**	$P2_1/m$	1.75	18.407	10.229	9.443	90	93.90	90	1774	*c*
**323**	$P2_1/m$	1.68	18.405	10.188	9.436	90	93.94	90	1765	*c*

Supplementary Table 3. Results of the Pawley fitting of the VTPXRDs recorded for **D1** (*h* = heating branch, *c* = cooling branch). Errors for given values a, b, c, β, and V correspond to last significant position. Space group was fixed for fitting according to literature known parent material property.

T [K]	space group	r_{wp}	a [Å]	b [Å]	c [Å]	α [°]	β [°]	γ [°]	V [Å^3]	branch
**323**	$P2_1/m$	1.65	18.626	10.273	9.556	90	93.19	90	1826	*h*
**343**	$P2_1/m$	1.62	18.612	10.313	9.559	90	93.23	90	1832	*h*
**363**	$P2_1/m$	1.70	18.610	10.359	9.563	90	93.26	90	1841	*h*
**383**	$P2_1/m$	1.97	18.611	10.391	9.565	90	93.17	90	1847	*h*
**403**	$P2_1/m$	2.14	18.611	10.427	9.567	90	93.10	90	1854	*h*
**423**	$P2_1/m$	2.14	18.603	10.483	9.569	90	93.20	90	1863	*h*
**443**	$P2_1/m$	1.98	18.565	10.564	9.571	90	93.60	90	1873	*h*
**453**	$P2_1/m$	1.96	18.587	10.583	9.568	90	93.62	90	1878	*h*
**463**	$P2_1/m$	1.88	18.596	10.595	9.571	90	93.63	90	1882	*h*
**473**	$P2_1/m$	1.90	18.613	10.603	9.572	90	93.62	90	1885	*h*
**483**	$P2_1/m$	1.44	18.615	10.612	9.576	90	93.78	90	1887	*h*
**493**	$P2_1/m$	1.50	18.634	10.633	9.574	90	93.72	90	1893	*h*
**503**	$P2_1/m$	1.44	18.657	10.628	9.580	90	93.77	90	1895	*h*
**513**	$P2_1/m$	1.48	18.679	10.628	9.579	90	93.63	90	1898	*h*
**503**	$P2_1/m$	1.44	18.660	10.625	9.579	90	93.75	90	1895	*c*
**493**	$P2_1/m$	1.56	18.640	10.615	9.575	90	93.71	90	1891	*c*
**483**	$P2_1/m$	1.50	18.615	10.612	9.576	90	93.75	90	1888	*c*
**473**	$P2_1/m$	1.48	18.625	10.606	9.576	90	94.00	90	1887	*c*
**463**	$P2_1/m$	1.46	18.604	10.602	9.571	90	93.93	90	1883	*c*
**453**	$P2_1/m$	1.53	18.585	10.591	9.575	90	94.21	90	1880	*c*
**443**	$P2_1/m$	1.46	18.568	10.566	9.571	90	94.25	90	1873	*c*
**423**	$P2_1/m$	1.50	18.532	10.520	9.566	90	94.30	90	1860	*c*
**403**	$P2_1/m$	1.58	18.515	10.476	9.562	90	94.12	90	1850	*c*
**383**	$P2_1/m$	1.55	18.539	10.419	9.550	90	93.78	90	1841	*c*
**363**	$P2_1/m$	1.56	18.546	10.364	9.543	90	93.63	90	1831	*c*
**343**	$P2_1/m$	1.56	18.556	10.318	9.534	90	93.45	90	1822	*c*
**323**	$P2_1/m$	1.54	18.580	10.268	9.527	90	93.36	90	1814	*c*

Supplementary Table 4. Results of the Pawley fitting of the VTPXRDs recorded for **D2** (*h* = heating branch, *c* = cooling branch). Errors for given values a, b, c, β, and V correspond to last significant position. Space group was fixed for fitting according to literature known parent material property.

T [K]	space group	r_{wp}	a [Å]	b [Å]	c [Å]	α [°]	β [°]	γ [°]	V [Å^3]	branch
**323**	$P2_1/m$	1.94	18.704	10.329	9.608	90	92.88	90	1854	*h*
**343**	$P2_1/m$	1.91	18.689	10.370	9.612	90	93.02	90	1860	*h*
**363**	$P2_1/m$	1.75	18.666	10.405	9.618	90	93.20	90	1865	*h*
**383**	$P2_1/m$	1.56	18.681	10.429	9.621	90	93.13	90	1872	*h*
**403**	$P2_1/m$	1.72	18.683	10.456	9.623	90	93.12	90	1877	*h*
**423**	$P2_1/m$	1.61	18.660	10.502	9.627	90	93.24	90	1884	*h*
**443**	$P2_1/m$	1.65	18.631	10.581	9.621	90	93.46	90	1893	*h*
**453**	$P2_1/m$	1.82	18.638	10.606	9.623	90	93.46	90	1899	*h*
**463**	$P2_1/m$	1.81	18.654	10.622	9.625	90	93.48	90	1904	*h*
**473**	$P2_1/m$	1.80	18.677	10.643	9.628	90	93.43	90	1910	*h*
**483**	$P2_1/m$	1.76	18.705	10.663	9.631	90	93.37	90	1918	*h*
**493**	$P2_1/m$	1.94	18.727	10.679	9.632	90	93.40	90	1923	*h*
**503**	$P2_1/m$	1.97	18.751	10.687	9.636	90	93.43	90	1928	*h*
**513**	$P2_1/m$	2.24	18.763	10.676	9.638	90	93.46	90	1927	*h*
**503**	$P2_1/m$	2.35	18.746	10.683	9.637	90	93.45	90	1926	*c*
**493**	$P2_1/m$	2.30	18.730	10.674	9.636	90	93.44	90	1923	*c*
**483**	$P2_1/m$	2.22	18.710	10.665	9.632	90	93.47	90	1919	*c*
**473**	$P2_1/m$	2.17	18.690	10.651	9.630	90	93.46	90	1914	*c*
**463**	$P2_1/m$	2.25	18.679	10.642	9.629	90	93.46	90	1911	*c*
**453**	$P2_1/m$	2.74	18.655	10.618	9.625	90	93.48	90	1903	*c*
**443**	$P2_1/m$	2.42	18.631	10.601	9.620	90	93.56	90	1896	*c*
**423**	$P2_1/m$	2.44	18.608	10.549	9.612	90	93.66	90	1883	*c*
**403**	$P2_1/m$	2.48	18.604	10.497	9.605	90	93.65	90	1872	*c*
**383**	$P2_1/m$	2.03	18.611	10.450	9.595	90	93.58	90	1862	*c*
**363**	$P2_1/m$	2.36	18.586	10.408	9.591	90	93.67	90	1851	*c*
**343**	$P2_1/m$	2.45	18.579	10.371	9.588	90	93.72	90	1844	*c*
**323**	$P2_1/m$	3.67	18.579	10.371	9.588	90	93.72	90	1844	*c*

Supplementary Table 5. Results of the Pawley fitting of the VTPXRDs recorded for **D3** (*h* = heating branch, *c* = cooling branch). Errors for given values a, b, c, β, and V correspond to last significant position. Space group was fixed for fitting according to literature known parent material property.

T [K]	space group	r_{wp}	a [Å]	b [Å]	c [Å]	α [°]	β [°]	γ [°]	V [Å^3]	branch
**323**	$P2_1/m$	2.27	18.528	10.305	9.542	90	93.88	90	1818	*h*
**343**	$P2_1/m$	2.30	18.534	10.342	9.548	90	93.88	90	1826	*h*
**363**	$P2_1/m$	2.06	18.532	10.371	9.553	90	93.88	90	1832	*h*
**383**	$P2_1/m$	1.94	18.554	10.404	9.554	90	93.75	90	1840	*h*
**403**	$P2_1/m$	1.87	18.563	10.435	9.556	90	93.72	90	1847	*h*
**423**	$P2_1/m$	1.88	18.548	10.478	9.561	90	93.85	90	1854	*h*
**443**	$P2_1/m$	1.86	18.531	10.502	9.558	90	94.01	90	1856	*h*
**453**	$P2_1/m$	1.91	18.539	10.504	9.561	90	94.02	90	1857	*h*
**463**	$P2_1/m$	1.91	18.553	10.507	9.562	90	94.04	90	1859	*h*
**473**	$P2_1/m$	1.89	18.567	10.501	9.564	90	94.03	90	1860	*h*
**483**	$P2_1/m$	1.97	18.580	10.501	9.565	90	94.04	90	1862	*h*
**493**	$P2_1/m$	2.06	18.601	10.500	9.569	90	94.02	90	1864	*h*
**503**	$P2_1/m$	2.21	18.619	10.501	9.572	90	94.00	90	1867	*h*
**513**	$P2_1/m$	2.63	18.641	10.515	9.573	90	94.01	90	1872	*h*
**503**	$P2_1/m$	2.81	18.617	10.511	9.574	90	94.05	90	1869	*c*
**493**	$P2_1/m$	2.90	18.597	10.504	9.574	90	94.12	90	1865	*c*
**483**	$P2_1/m$	2.96	18.578	10.502	9.574	90	94.16	90	1863	*c*
**473**	$P2_1/m$	2.87	18.556	10.500	9.571	90	94.16	90	1860	*c*
**463**	$P2_1/m$	2.88	18.539	10.500	9.570	90	94.20	90	1858	*c*
**453**	$P2_1/m$	2.78	18.518	10.502	9.568	90	94.22	90	1856	*c*
**443**	$P2_1/m$	2.73	18.504	10.503	9.565	90	94.22	90	1854	*c*
**423**	$P2_1/m$	2.64	18.485	10.497	9.558	90	94.23	90	1850	*c*
**403**	$P2_1/m$	2.66	18.473	10.484	9.551	90	94.25	90	1845	*c*
**383**	$P2_1/m$	2.72	18.461	10.447	9.542	90	94.22	90	1835	*c*
**363**	$P2_1/m$	2.61	18.483	10.388	9.536	90	94.08	90	1826	*c*
**343**	$P2_1/m$	2.50	18.511	10.326	9.530	90	93.94	90	1817	*c*
**323**	$P2_1/m$	2.52	18.486	10.293	9.520	90	93.96	90	1807	*c*

S7 Thermal expansion calculation

Cell parameter obtained from Pawley fits (S6) were used as input for PASCal to determine principal axis strain and coefficients of thermal expansion (CTE).[8] Due to the observed second order phase transition CTEs were calculated from separate input data sets for low temperature (up to 403 K). Principal directions are attributed to the approximate axis of projection on [uvw] for best comparison of low and high temperature CTEs.

Supplementary Table 6. Principal axis directions, their approximate projection onto [uvw] and thermal expansion coefficients α for 323-403 K with standard deviation.

		α [M/K]	Δα [M/K]	u	v	w	appr. axis
**D0**	**X_1**	-82.0821	9.0308	0.703	0	0.7112	[101]
	**X_2**	60.9056	3.1341	-0.2341	0	0.9722	[001]
	**X_3**	**235.2796**	**8.4081**	**0**	**1**	**0**	**[010]**
	**V**	216.4853	11.5873				
**D1**	**X_1**	-10.3286	4.05	0.8028	0	0.5963	[101]
	**X_2**	16.7716	0.9286	-0.1681	0	0.9858	[001]
	**X_3**	**188.1439**	**3.7886**	**0**	**1**	**0**	**[010]**
	**V**	195.0737	2.3586				
**D2**	**X_1**	-28.5696	12.603	0.6458	0	0.7635	[101]
	**X_2**	32.8041	6.263	-0.2831	0	0.9591	[001]
	**X_3**	**151.8102**	**7.6035**	**0**	**1**	**0**	**[010]**
	**V**	156.3307	1.7664				
**D3**	**X_1**	4.0495	1.2498	0.9933	0	0.1153	[100]
	**X_2**	40.9069	1.81	0.004	0	1	[001]
	**X_3**	**156.0797**	**2.3603**	**0**	**1**	**0**	**[010]**
	**V**	202.1619	2.4249				

S8 Electron microscopy

Scanning transmission electron microscopy (STEM) micrographs with energy dispersive X-ray spectroscopy (EDS) elemental mappings were recorded with a JEM-ARM200F “NEOARM” microscope from JEOL (Germany) GmbH with a cold FEG electron source operated at 200 kV. Samples were prepared by depositing a drop of the solid dispersed in ethanol onto carbon-coated copper grids (200 mesh) and dried in air. The electron tomography was carried out in a 2 or 3° step using the TEMography™ software for both recording and 3D-image reconstruction.

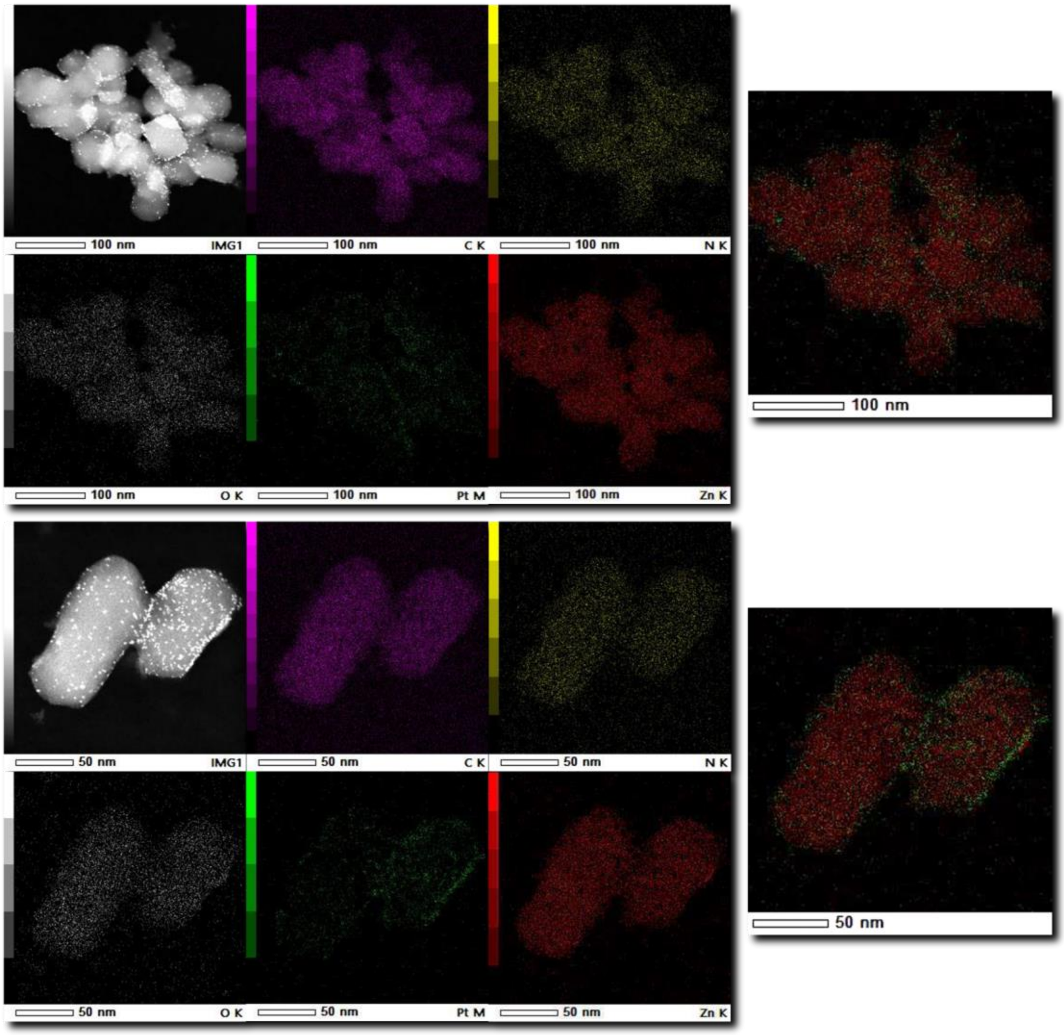

Supplementary Figure 7: STEM-EDS micrographs of two different **D1** crystallite formations. Darkfield TEM micrograph (top left) and EDS elemental mapping (top mid, top right, bottom row) shown with respect to single elements. Larger image (right) with Pt and Zn overlay (contrast adjusted for visibility). Purple: C, yellow: N, white: O, green: Pt, red: Zn.

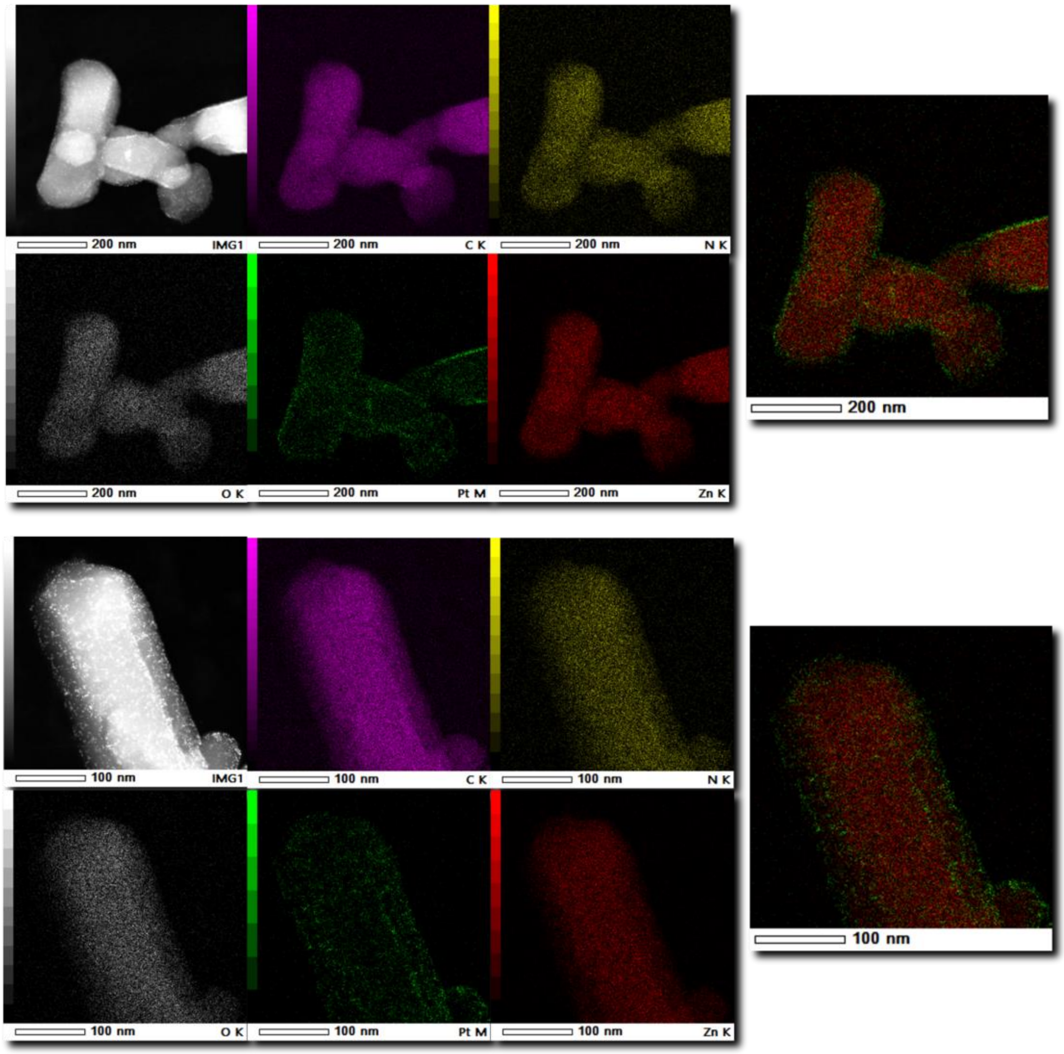

Supplementary Figure 8: STEM-EDS micrographs of two different **D2** crystallite formations. Darkfield TEM micrograph (top left) and EDS elemental mapping (top mid, top right, bottom row) shown with respect to single elements. Larger image (right) with Pt and Zn overlay. Purple: C, yellow: N, white: O, green: Pt, red: Zn.

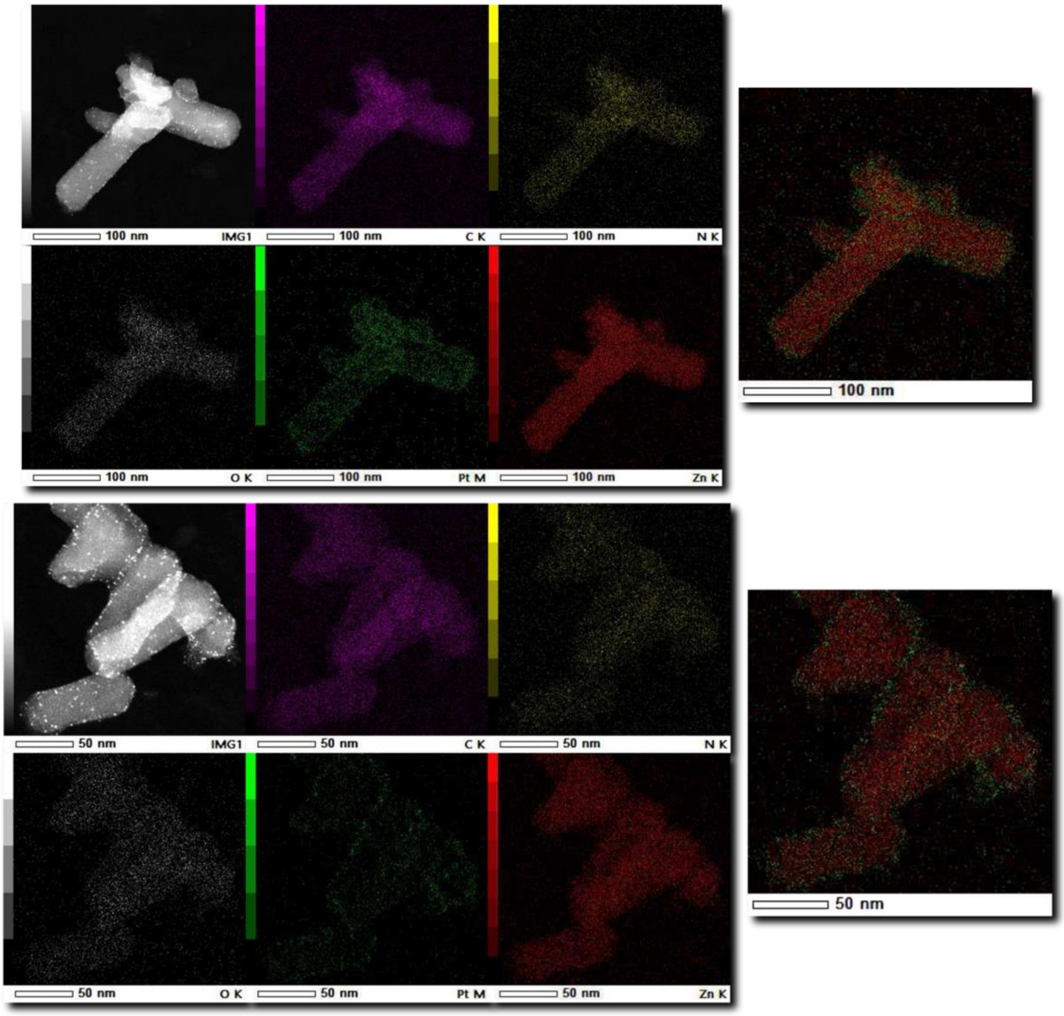

Supplementary Figure 9: STEM-EDS micrographs of two different **D3** crystallite formations. Darkfield TEM micrograph (top left) and EDS elemental mapping (top mid, top right, bottom row) shown with respect to single elements. Larger image (right) with Pt and Zn overlay (contrast adjusted for visibility). Purple: C, yellow: N, white: O, green: Pt, red: Zn.

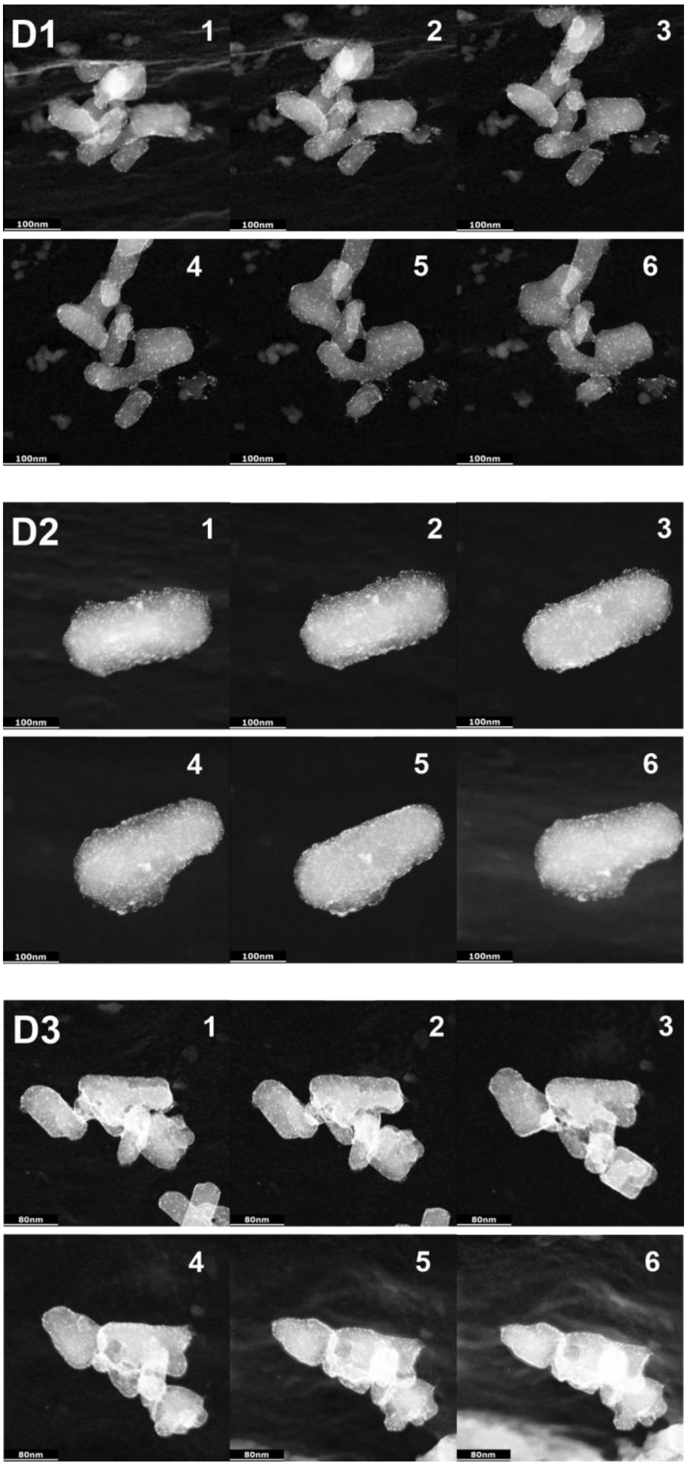

Supplementary Figure 10: Six selected examples of each tilt-series for **D1**, **D2**, and **D3** (out of ca. 40-60 per sample).

S9 Supplementary references

1. Henke, S., Schneemann, A., Wütscher, A. & Fischer, R. A. Directing the breathing behavior of pillared-layered metal-organic frameworks via a systematic library of functionalized linkers bearing flexible substituents. *J. Am. Chem. Soc.* **134,** 9464–9474; 10.1021/ja302991b (2012).

2. Schneemann, A., Henke, S., Schwedler, I. & Fischer, R. A. Targeted manipulation of metal-organic frameworks to direct sorption properties. *ChemPhysChem* **15,** 823–839; 10.1002/cphc.201300976 (2014).

3. Vervoorts, P. *et al.* Configurational Entropy Driven High-Pressure Behaviour of a Flexible Metal-Organic Framework (MOF). *Angew. Chem.* **60,** 787–793; 10.1002/anie.202011004 (2021).

4. Henke, S., Schneemann, A. & Fischer, R. A. Massive Anisotropic Thermal Expansion and Thermo-Responsive Breathing in Metal-Organic Frameworks Modulated by Linker Functionalization. *Adv. Funct. Mater.* **23,** 5990–5996; 10.1002/adfm.201301256 (2013).

5. ZareKarizi, F., Joharian, M. & Morsali, A. Pillar-layered MOFs: functionality, interpenetration, flexibility and applications. *J. Mater. Chem. A* **6,** 19288–19329; 10.1039/C8TA03306D (2018).

6. Henke, S., Schmid, R., Grunwaldt, J.-D. & Fischer, R. A. Flexibility and sorption selectivity in rigid metal-organic frameworks: the impact of ether-functionalised linkers. *Chemistry* **16,** 14296–14306; 10.1002/chem.201002341 (2010).

7. Lee, W.-J. *et al.* Synthesis of highly dispersed Pt nanoparticles into carbon supports by fluidized bed reactor atomic layer deposition to boost PEMFC performance. *NPG Asia Mater* **12**; 10.1038/s41427-020-0223-x (2020).

8. Cliffe, M. J. & Goodwin, A. L. PASCal : a principal axis strain calculator for thermal expansion and compressibility determination. *J. Appl. Crystallogr.* **45,** 1321–1329; 10.1107/S0021889812043026 (2012).
