# Supplementary material for: Reduced thermal expansion by surface-mounted nanoparticles in a pillared-layered metal-organic framework
Source: Commun Chem. 2022 Dec 22;5:177. doi: 10.1038/s42004-022-00793-2 (PMC9814677; doi:10.1038/s42004-022-00793-2)
Supplement: Supplementary file 3 — Description of Additional Supplementary Files [file 42004_2022_793_MOESM3_ESM.pdf]

Description of Additional Supplementary Files

File name: Supplementary Data 1

Description: Adsorption information files for each isotherm.

File name: Supplementary Video 1

Description: : STEM tilt-series example.

File name: Supplementary Video 2

Description: : Reconstruction from electron tomography.
